# Time for Dementia: innovation in healthcare student dementia education

**DOI:** 10.1002/alz70861_108053

**Published:** 2025-12-23

**Authors:** Stephanie Daley, Molly Hebditch, Yvonne Feeney, Sube Banerjee

**Affiliations:** ^1^ Brighton and Sussex Medical School, Brighton UK; ^2^ University of Nottingham, Nottingham UK

## Abstract

**Session overview:**

**Aims**: The future healthcare workforce requires the skills, attitudes, and empathy to better meet the needs of those living with dementia. Time for Dementia is an educational programme in which healthcare students from a range of professional groups (medicine, nursing, allied health professionals) undertake a number of home visits to a family (person with dementia and their family carer) over a two‐year period. The programme also brings students together with the wider network of participating families to share experiences and reflect on learning. The aim of this study was to evaluate the impact of Time for Dementia on student attitudes, knowledge and empathy towards dementia.

**Methods:**

A mixed methods longitudinal cohort study was conducted between 2014‐ 2021. Qualitative interviews and focus groups were undertaken with 93 healthcare students. Measures of dementia knowledge, attitudes and empathy were administered to students at five universities in the south of England before and after they completed the Time for Dementia programme. Data were also collected at equivalent time points for a control group of students who had not taken part in the programme. Outcomes were modelled using multilevel linear regression models.

**Results:**

2,700 intervention group students and 562 control group students consented to participate in the research. Students undertaking the Time for Dementia programme had higher levels of knowledge and positive attitudes at follow‐up compared to equivalent students who did not undertake the programme. Key themes identified from the qualitative analysis highlighted relational learning, insight and understanding, challenge (of) attitude and stigma and enhanced dementia practice. Our findings indicate a positive relationship between the number of home visits undertaken and increasing dementia knowledge and attitudes.

**Conclusions:**

The results suggest that the Time for Dementia programme is effective in improving the knowledge and attitudes of healthcare students across different professional groups and universities.

